# Homœopathy Proven by Koch

**Published:** 1891-11

**Authors:** 


### HOMŒOPATHY PROVEN BY KOCH.

Our neighbors have been rejoicing for months that the truth of Homœopathy has been proven by the new cure for tuberculosis. Hardly a journal in the land but has had something to say about it, and some have said a good deal. Of course it proved the truth of their law, *similia similibus.* It went further, and proved the value of infinitesimals. And still further, it proved the value of *nosodes*, the dirty part of Homœopathy.

And now our sound homœopathists may exclaim, "The Lord save me from my friends; I can take care of my enemies." The entire Koch business has proven a failure; not one patient has been cured, but scores have died from it. Is Homœopathy to be measured by this standard? It may be *similia;* it is certainly a very vile *nosode*, and hundreds have had the tubercular bacillus distributed in their tissues by it, and others have suffered from the effects of the most poisonous ptomaine ever known. How does the homœopathic *nosode* business compare with this?

As you look the field over, my friends, do you really think you have made anything by appropriating *regular* thunder? I imagine that you had better stick to the legitimate, and to that you know. When you try to become "scientific" by riding a bacterium, or appropriating a *regular* nosode, you are likely to make a failure. It is not my province to advise you, but many of you are clever men and co-workers, and I cannot help saying, stick to the truths you know, and don't toady to the "regulars." —*The Eclectic Medical Journal, June,* 1891.

